# Long-term monthly mesenchymal stromal cell therapy for advanced chronic lung allograft dysfunction: a single-centre safety and feasibility cohort study

**DOI:** 10.3389/fmed.2026.1942517

**Published:** 2026-09-14

**Authors:** Sara Lettieri, Maria Antonietta Avanzini, Domenica Federica Briganti, Sara Bozzini, Eleonora Bozza, Elisa Lenta, Valentina Vertui, Francesca Mariani, Angelo Guido Corsico, Klodiana Mucaj, Mariachiara Crescenzi, Annalisa De Silvestri, Patrizia Comoli, Mirko Belliato, Federica Meloni

**Affiliations:** 1 Pneumology Unit, IRCCS San Matteo Hospital Foundation, Pavia, Italy; 2 Department of Internal Medicine and Therapeutics, University of Pavia, Pavia, Italy; 3 SSD Cell Factory and Center for Advanced Therapies, IRCCS San Matteo Hospital Foundation, Pavia, Italy; 4 Pediatric Hematology/Oncology Unit, IRCCS San Matteo Hospital Foundation, Pavia, Italy; 5 Second Department of Anesthesia and Intensive Care, IRCCS San Matteo Hospital Foundation, Pavia, Italy; 6 Department of Medical and Surgical Sciences (DiMEC), University of Bologna, Bologna, Italy; 7 Biometry Unit, IRCCS San Matteo Hospital Foundation, Pavia, Italy; 8 Department of Translational Medicine (DIMET), University of Piemonte Orientale UniUPO and IRCCS SS. Antonio e Biagio e Cesare Arrigo, Alessandria, Italy

**Keywords:** bronchiolitis obliterans syndrome, chronic lung allograft dysfunction, lung transplantation, mesenchymal stromal cells, regenerative therapy

## Abstract

**Introduction:**

Chronic lung allograft dysfunction (CLAD) remains the leading cause of late morbidity and mortality after lung transplantation, with limited therapeutic options. Mesenchymal stromal cells (MSCs) have immunomodulatory and regenerative properties that may have therapeutic potential in CLAD.

**Methods:**

We evaluated the long-term safety and feasibility of repeated intravenous MSC administration in patients with advanced CLAD refractory to standard treatments. Six lung transplant patients with progressive moderate-to-severe CLAD received monthly infusions of allogeneic, bone marrow–derived, HLA-unmatched MSCs (1 × 10⁶ cells/kg). Safety was assessed by survival and infection incidence. Pulmonary function (FEV₁, FVC) and laboratory parameters were monitored longitudinally. Interrupted time-series analysis was used to evaluate changes in functional trends.

**Results:**

A total of 251 MSC infusions were administered during the study period, with follow-up extending up to 58 months. All patients completed at least 24 months of follow-up. Survival at 24 months was 100%; one patient later died from pulmonary infection after being listed for retransplantation. MSC therapy was well tolerated, with no infusion-related toxicity, serious treatment-related adverse events or statistically significant increase in infection risk. While immediate changes in FEV₁ or FVC were not observed, the rate of functional decline significantly decreased post-treatment, suggesting a potential attenuation of functional decline. Laboratory parameters remained stable throughout follow-up, with no evidence of systemic toxicity.

**Conclusion:**

Repeated long-term intravenous administration of allogeneic bone marrow- derived MSCs in patients with advanced, treatment-refractory CLAD appears feasible, safe, and generally well tolerated. Although no statistically significant increase in respiratory infection incidence was observed, the study was underpowered to exclude a clinically meaningful increase in infection risk. In our cohort, lung function decline appeared to stabilize in some patients following MSC initiation over time; however, the observed changes in lung function trajectories are exploratory and should not be interpreted as evidence of therapeutic efficacy. Controlled clinical trials are needed to clarify the potential role of MSC therapy and to define optimal treatment strategies and patient selection.

## 1 Introduction

Lung transplantation (Ltx) is the only treatment available for several end-stage respiratory diseases, including chronic obstructive pulmonary disease (COPD), interstitial lung diseases (ILDs), pulmonary hypertension, and cystic fibrosis. Despite its life-saving potential, the median survival after lung transplant remains below 7 years (1). Chronic lung allograft dysfunction (CLAD) is the main factor limiting long term survival (2). It results from repeated immunological and non-immunological insults, that damage the epithelial barrier, impair epithelial regeneration, and reduce the secretion of protective proteins (3) which ultimately trigger excessive fibrotic repair associated to a progressive and irreversible graft function deterioration. CLAD is currently regarded as an umbrella definition encompassing several presentations, with two main phenotypes recognized, named bronchiolitis obliterans syndrome (BOS) and restrictive allograft syndrome (RAS). Other well defined but less common phenotypes are the mixed, with coexisting features of BOS and RAS, and the undefined one (2, 4). Current therapeutic options for CLAD include azithromycin (5), montelukast (6), lymphodepletion with anti-thymocyte globulins (ATG) or alemtuzumab (7, 8) and immune-regulation by means of total lymphoid irradiation (TLI) or extracorporeal photopheresis (ECP) (9–15). Most of these approaches have been reported, in retrospective studies, to stabilize graft function or slow the rate of decline in up to 50%–60% of treated cases (12). Nevertheless, retransplantation remains the last therapeutic option for progressive CLAD but is associated to worse prognosis than the primary lung transplant and is generally reserved for carefully selected, younger CLAD patients mainly with the obstructive phenotype (16). In this context, the evaluation of new possible therapeutic approaches in CLAD is crucial.

Mesenchymal stromal cells (MSCs) have gained significant attention in the field of regenerative medicine due to their potential to promote tissue repair, modulate immune responses, and reduce inflammation. These effects are exerted through a wide *secretoma* comprised by cytokines, growth factors, microRNAs, and extracellular vesicles, acting in paracrine way (17). Lung resident MSCs have a protective effect by promoting epithelial (18) and endothelial repair (19, 20) and polarizing macrophages towards an immunosuppressive M2-like phenotype for the restoration of tissue homeostasis (21). If administered systemically for therapeutic purposes, they can home and accumulate to the sites of damage, with a high rate of pulmonary accumulation as first passage organ, thus providing an advantage for the potential treatment of chronic inflammatory pulmonary diseases (22).

Several clinical studies have shown intravenous MSCs safety and potential efficacy in the treatment of both Covid and non- Covid acute respiratory distress syndrome (22–24).

Some experimental *in vitro* and *ex vivo* evidences suggest a role of MSCs in the regulation of alloimmune responses in the context of solid organ and bone marrow transplantation (25). In lung transplantation, MSCs have been administered to the donor lungs to prevent the ischemia-reperfusion injury (IRI) and the consequent primary graft dysfunction (PGD) (25). Bone marrow–derived MSCs (BM-MSCs) have also been studied as a novel therapeutic strategy for BOS post lung transplantation. In preclinical models, including heterotopic tracheal transplantation in rats, MSCs delivered by endotracheal or intravenous routes (IV) reduced fibrotic obliteration in bronchioles, decreased airway fibrosis and edema, and promoted reparative processes (26). In the first-in-human phase trial (NCT01175655), allogeneic BM-MSCs were administered to patients with moderate-to-severe BOS. The treatment was safe and well tolerated; most recipients experienced stabilization of lung function (FEV₁/FVC), with no serious infusion-related adverse events (27). A subsequent phase II randomized trial, ASSIST-CLAD, confirmed safety and feasibility of IV MSC infusions in new-onset CLAD cases, although it did not demonstrate improvements in progression-free survival or lung function decline compared with placebo (28). Similarly, Erasmus et al. reported that low-dose of IV BM-MSC infusions were well tolerated in patients with established obstructive CLAD and appeared to slow the decline of FEV₁ over one year compared with pre-treatment trajectories (29). However, previous clinical studies on BOS, have employed short treatment schedules and heterogeneous dosing regimens (number of infused cells per kg of body weight). Consequently, uncertainties remain regarding the safety, tolerability, and potential clinical effects of prolonged low-dose MSCs administration, similar to treatment strategies adopted for Graft Versus Host Disease (GVHD) (30, 31).

Therefore, the primary aim of this study was to evaluate the feasibility and safety of repeated intravenous administration of allogeneic, third-party bone marrow–derived, human leukocyte antigen (HLA)–unmatched BM-MSCs in patients with advanced CLAD who have not responded to multiple lines of therapy. We have measured survival, incidence of lung infections and other serious adverse effects. Secondary exploratory objectives were to assess longitudinal changes of functional parameters, expressed as forced expiratory volume in one second (FEV₁) and forced vital capacity (FVC), as well as trends in laboratory findings before and after MSC administration.

## 2 Materials and methods

### 2.1 Study design

Between November 2020 and May 2023 we consecutively enrolled six lung-transplant recipients followed up at IRCCS San Matteo Hospital Foundation in Pavia and diagnosed with CLAD grade 3–4, according to the International Society for Heart and Lung Transplantation (ISHLT) guidelines (2), who had clinical or functional deterioration despite optimization of immunosuppressive treatment, ECP, azithromycin, and montelukast.

On a compassionate-use basis, patients received monthly infusions of allogeneic BM-MSCs at a dose of 1 × 10⁶ cells/kg body weight. Patients with a history of thromboembolic events, active infections, or pulmonary colonization by difficult to treat pathogens (i.e., extended drug resistant bacteria, fungi) were excluded. The study was approved by the local Ethics Committee and conducted in accordance with the Declaration of Helsinki.

Initially, patients were approved to receive MSC administration for 6–9 months. Given the favourable outcomes, authorization to continue treatment was subsequently granted, and patients remained on therapy.

### 2.2 MSC preparation

BM-MSCs were generated from bone marrow aspirates obtained from screened healthy third-party hematopoietic stem cell donors under European Good Manufacturing Practice (GMP) conditions at the GMP Cell Factory of the Fondazione IRCCS Policlinico San Matteo (Pavia, Italy). Mononuclear cells were isolated by Ficoll density-gradient centrifugation and expanded in platelet lysate-supplemented Dulbecco's Modified Eagle Medium until passage 4, which constituted the final clinical product. Before release, each MSC batch underwent predefined biological, microbiological, and molecular quality control testing. Product identity was confirmed by short tandem repeat analysis, while purity was assessed by morphology, viability (≥70%), and immunophenotypic characterization according to the International Society for Cell and Gene Therapy criteria (CD73, CD90, and CD105 ≥ 95%; combined expression of CD34, CD45, CD19, and HLA-DR ≤5%) (32). Safety testing included aerobic and anaerobic bacterial and fungal cultures, endotoxin testing, and mycoplasma PCR, all of which were required to be negative before clinical release. After thawing, MSCs were diluted in saline solution + 5% human albumin under a class A laminar hood and intravenously infused within one hour at the dose of 1 × 10^6^/Kg body weight. Infusions were delivered slowly over 10 min through a large peripheral vein. Vital signs, including heart rate, blood pressure, and peripheral oxygen saturation, were continuously monitored for one hour after each infusion. Physical examination, laboratory tests, donor-specific anti-HLA antibody (DSA) assessment, and pulmonary function tests (PFTs) were performed before each MSC administration. Respiratory infections were identified through review of the medical records and were defined as clinically significant lower respiratory tract infections requiring antimicrobial treatment and/or supported by microbiological confirmation. All infectious episodes occurring during the 36 months before MSC initiation and throughout follow-up were recorded.

### 2.3 Statistics

Interrupted time-series analysis was used to assess the effect of an intervention over time, focusing on changes and change in trends before and after the index date (start of treatment). To model this, we applied splines to introduce a knot at the index date and fitted a mixed linear model with random intercepts (patients) and random slopes for time-related variables. This approach allowed us to estimate the pre-intervention trend, the immediate change in outcome after the intervention, and the difference in slopes before and after the index date.

## 3 Results

### 3.1 Study population

The study included six bilateral lung transplant recipients (4 males and 2 females). The underlying diseases were COPD (*n* = 1), ILDs (*n* = 3), among which 2 were idiopathic pulmonary fibrosis (IPF) and 1 was fibrotic hypersensitivity pneumonitis (fHP), obliterative bronchiolitis (OB) in rheumatoid arthritis (*n* = 1), re-transplant for BOS (*n* = 1). All patients received a triple maintenance immunosuppressive treatment consisting of a calcineurin inhibitor, corticosteroid and a cell cycle inhibitor, except for one patient, who received only tacrolimus and steroids because of severe side effects with the third agent. CLAD was diagnosed, according to the ISHLT guidelines (2). Three patients had a BOS, three had a mixed phenotype.

At baseline, DSAs were tested and resulted negative, except patient n°5 who had persistent *de novo* anti class II DSA (HLA DR3; Mean Fluorescence Intensity 1,000–3,000 by Luminex single-antigen bead assay), and had received, 6 months before CLAD diagnosis, a treatment for a possible antibody-mediated rejection according to ISHLT guidelines (33), treated with a high dose steroid course, plasma exchange, and high dose intravenous immunoglobulins.

At CLAD onset, all patients received Azithromycin and Montelukast together with optimization of immunosuppression, including a shift from mycophenolate to everolimus when possible. At progression to grade 2 CLAD, long term offline ECP was proposed. At failure of ECP recipients were enrolled in the study.

The mean age at the start of MSC treatment was 57.6 years (SD ± 8.42) and the median follow up from lung transplantation to MSC initiation was 10.5 years (±IQR 13.75). Further clinical and demographic features of the patients are summarized in the Table 1.

**Table 1:** Clinical and demographic features of the patients.

Patient	Sex	Indication for lung tx	Year of lung tx	Time from tx to CLAD (years)	CLAD stage/phenotype	Immunosuppressive treatment at the beginning of MSCs therapy	Age at MSCs treatment	Time from tx to MSCs (years)	Month and year of MSCs initiation	Frequent bronchial exacerbations (>= 3/Year)	Humoral rejection
Patient 1	M	Fibrotic hypersensitivity pneumonitis	2016	3	IV, BOS	Tacrolimus, everolimus, prednisone	47	4	November 2020	yes	no
Patient 2	M	Obliterative bronchiolitis in rheumatoid arthritis	2005	10	IV, BOS	Tacrolimus, mycophenolate, prednisone	71	16	February 2021	yes	no
Patient 3	F	Re-transplant for BOS	2000	12	IV, BOS	Tacrolimus, prednisone	48	22	July 2022	yes	no
Patient 4	M	IPF	2016	4,5	III, mixed	Tacrolimus, everolimus, prednisone	62	6	August 2022	no	no
Patient 5	F	COPD	2019	3	IV, mixed	Cyclosporine, everolimus, prednisone	62	3,5	December 2022	no	yes
Patient 6	M	IPF	2007	11	IV, mixed	Tacrolimus, everolimus, prednisone	56	15	May 2023	yes	no

Tx, transplant; MSCs, mesenchymal stromal cells; M, male; F, female; IPF, idiopathic pulmonary fibrosis; COPD, chronic obstructive pulmonary disease; CLAD, chronic lung allograft dysfunction; BOS, bronchiolitis obliterans syndrome.

### 3.2 Safety

Overall a total of 251 monthly infusions were administered to the 6 patients enrolled (median treatment 37.5 months (IQR 22.5) with a minimum follow up of 28 months and a maximum follow up of 58 months.

Even taking into account heterogeneity in the length of MSC treatment and follow up, all patients completed a minimum of 24 months of follow up. Survival after 24 months of monthly MSCs infusion was 100%. One patient was listed for lung retransplantation after 34 months of MSC treatment for progression of CLAD and died 3 months later due to a severe pulmonary infection. The other five patients (83.3%) are still alive and under monthly treatment with MSCs (median treatment 37 months, IQR 25) with a minimum follow up of 28 months and a maximum follow up of 58 months at the time of present analysis. The incidence of lung infections did not significantly increase post-treatment (IRR = 1.33, 95% CI: 0.67–2.61; *p* = 0.41). However, the wide confidence interval reflects the limited precision of the estimate and does not exclude a clinically meaningful increase in infection risk. Overall, no clear infectious safety signal emerged during follow-up. Four patients (67%) experienced at least one respiratory infection during the study period (Table 2). In particular, Patient 2, who had experienced no respiratory infections during the 36-month pre-treatment period, developed three respiratory infections after MSC initiation. These episodes occurred over a prolonged follow-up period and were successfully managed with standard antimicrobial therapy without permanent discontinuation of MSC treatment. Globally, treatment with MSCs was well tolerated. No alterations of vital signs after infusion and no serious adverse events attributable to MSCs were recorded, one patient (17%) reported dizziness and one patient (17%) had episodes of metrorrhagia requiring treatment with gonadotropin-releasing hormone agonists. No fever, anaphylactic reactions, or thromboembolic events were reported.

**Table 2:** Spectrum of infections before and after initiation of MSCs, considering the period from 36 months before the MSC treatment to the last follow up (IRR = 1.33, 95% CI: 0.67–2.61; *p* = 0.41).

Patient	Number infections pre-MSC	Number infections post-MSC	Pathogens pre- MSC	Pathogens post-MSC
Patient 1	7	6	Pseudomonas aeruginosa, Aspergillus, Moraxella catarrhalis	Pseudomonas aeruginosa, Aspergillus, RSV
Patient 2	0	3		Pseudomonas aeruginosa, Haemophilus influenzae, Aspergillus
Patient 3	4	8	Pseudomonas aeruginosa, Staphylococcus aureus	Aspergillus, Klebsiella pneumoniae, Serratia marcescens, Staphylococcus aureus, Enterobacter cloacae
Patient 4	1	0	Aspergillus	
Patient 5	1	3	SARS-CoV-2	Staphylococcus epidermidis
Patient 6	2	0	Mycobacterium chimerae	

### 3.3 Lung function

From a functional perspective, there were no statistically significant changes of FEV1 (coefficient −0.031, 95% CI: −0.15; 0.09, *p* = 0.6) and FVC (coefficient −0.038,95% CI: −0.16; 0.09, *p* = 0.55) before and after treatment. However, interestingly, the slope of decline significantly decreased over time (FEV₁ *β* = –0.0189, *p* = 0.003; FVC *β* = –0.0139, *p* = 0.008). These findings indicate an apparent attenuation in the rate of lung function decline after MSC initiation. However, changes in lung function trajectories observed after MSC initiation should be considered exploratory and hypothesis-generating as the study design and the small sample size preclude conclusions regarding therapeutic efficacy. The trend of FEV1 and FVC over time is reported in the Figure 1.

**Figure 1:**
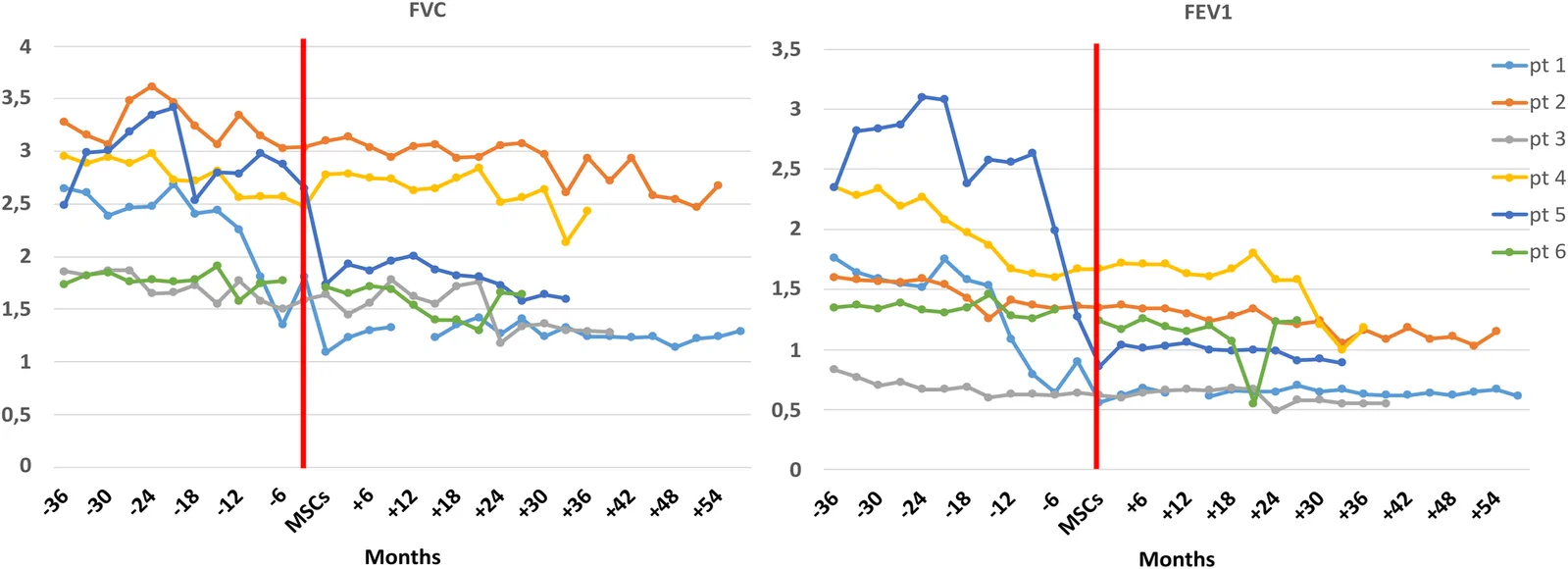
Trend of FEV1 and FVC before and after MSCs treatment in the six patients.

### 3.4 Blood parameters

No significant changes over the whole treatment were detected in the following variable values hemoglobin, leucocyte and platelet counts, prothrombin time (PT), international normalized ratio (INR), creatinine, potassium, liver enzymes, C-reactive protein (CRP), and lactate dehydrogenase (LDH), supporting the absence of a systemic toxicity of this therapeutic approach (Table 3). A near-significant rise in Calcium levels was detected post-therapy (IRR = 3.04, 95% CI: 0.9- 9.5, *p* = 0.057). Although it could theoretically indicate an effect of MSC infusion, it is more likely to reflect biological variability given the wide confidence interval and lack of correlated clinical deterioration. No additional *de novo* DSAs were detected, and no episodes of AMR occurred during MSC treatment.

**Table 3:** Median of blood parameters measured monthly 36 months before and after 24 months of MSCs administration.

Blood parameter	Median pre-infusion (IQR)	Median post-infusion (IQR)	Incidence rate ratio (IRR)	Standard deviation (SD)	*P*-value
Hemoglobin (gr/dL)	14 (11.9–14.7)	14.3 (12.7–15.3)	1.0	0.2	0.9
White blood cells (cells/mmc)	7.12 (6.3–8.91)	8.25 (7.04–9.42)	3.8	3	0.09
Platelets (cells/mmc)	198 (178–223)	231 (208–246)	0.65	0.37	0.47
Creatinine	0.99 (0.91–1.31)	1.01 (0.9–1.31)	1.19	0.38	0.59
Potassium	3.96 (3.72–4.33)	3.9 (3.8–4.2)	1.0	0.1	0.99
Calcium	9.2 (8.9–9.6)	9.3 (8.7–9.6)	3.05	1.78	0.05
LDH	189 (169–216)	208 (180–228)	1.16	0.39	0.43
ALT	16 (13–19)	16.5 (14–20)	1.08	1,08	0.93
GGT	13 (13–23.75)	19.5 (15–30)	1.05	1.05	0.9
CRP	0.21 (0.06–0.54)	0.195 (0.08–0.6)	1.09	0.35	0.78
PT	101 (90–109)	104 (90,75–118)	1.0	0.3	0.9
INR	0.98 (0.92–1.01)	0.97 (0.92–1.01)	0.7	0.47	0.6

## 4 Discussion

CLAD remains the major barrier to long-term survival after lung transplantation and, despite great advances in the management of lung transplant recipients, we still lack effective disease-modifying treatments. Its pathogenesis involves recurrent immunologic and non-immunologic injury, impaired epithelial repair, persistent inflammation, and progressive fibro-proliferation, ultimately resulting in irreversible decline in graft function (34). Current therapeutic strategies, including azithromycin, leukotriene receptor antagonists, intensified immunosuppression, ECP, and TLI, may offer transient stabilization in selected patients, but the ability to halt disease progression has not been consistently demonstrated. In this context, the use of a regenerative, immunomodulatory treatment can be an attractive approach.

MSCs are able to modulate immune cell proliferation, activation, and effector function, inhibit the expression of pro-inflammatory cytokines, promoting alveolar epithelial and endothelial repair and restore the tissue homeostasis through paracrine mechanisms. Interestingly, when they are infused intravenously, they remain partially entrapped, producing the “pulmonary first-pass effect” (35). This phenomenon is especially advantageous for lung-directed therapies as it increases the likelihood that MSCs reach areas of active injury, mitigating inflammation and facilitating endogenous repair processes (36).

Preclinical models and early human studies in acute lung injury, ischemia–reperfusion damage, idiopathic pulmonary fibrosis, graft-versus-host disease after hematopoietic stem cell transplantation and chronic rejection (22–24, 37–46) have demonstrated a favorable safety profile and biological activity. Early phase I studies in BOS further supported the feasibility of MSC-based therapies in lung transplant recipients (27, 29).

Notably, Chambers et al. reported a marked decline in the rate of lung function deterioration, with FEV₁ decline slowing from 120 mL/month pre-treatment to 30 mL/month post-allogeneic BM derived MSC therapy in 10 CLAD patients who received twice-weekly infusions (2 × 10^6^/kg for each infusion) over a short treatment period of two weeks (27). Similarly, Erasmus et al. documented stabilization of lung function over a 12-month period in 13 patients receiving MSCs in two administrations (29). However, treatment schedules and cell doses varied significantly across the studies, and most protocols involved short treatment courses and relatively limited follow-up. More recently, the randomized placebo-controlled ASSIST-CLAD trial confirmed the feasibility and safety of intravenous BM-MSC administration in 59 patients with new-onset CLAD but did not demonstrate significant improvements in progression-free survival or lung function decline compared with placebo (28). However, the treatment schedule was limited to four administrations in two weeks.

To our knowledge, our experience represents the first report describing prolonged monthly MSC administration in patients with advanced CLAD, with more than 250 infusions delivered and follow-up extending beyond two years. Our findings extend the available safety data by showing that prolonged monthly administration of HLA-unmatched BM-MSCs is feasible and generally well tolerated, even in patients with advanced CLAD refractory to multiple previous treatment strategies, including ECP. Importantly, repeated administration of HLA-unmatched MSCs did not result in significantly increased susceptibility to infections or other clinically relevant adverse events, and no infusion-related toxicity was observed during the study period. Moreover, MSC administration did not appear to induce new donor-specific antibodies, further supporting the immunological safety of this approach.

During the study period, all patients were alive 2 years after MSC initiation. Large European centres have reported lower 2-year survival rates in patients with advanced CLAD (16). However, these studies included different patient populations and cannot be considered directly comparable to our highly selected single-centre compassionate-use cohort.

From a functional perspective, no immediate improvement in lung function was observed after treatment initiation. Nevertheless, the trajectory of functional decline appeared to attenuate over time in our cohort, with a significant reduction in the slope of FEV₁ and FVC decline after MSC administration. This observation differs from the findings of the randomized ASSIST-CLAD trial, which did not demonstrate a significant effect of MSC therapy on lung function decline. Several factors may account for this discrepancy, including differences in patient population (advanced treatment-refractory CLAD versus new-onset CLAD), treatment regimen (prolonged monthly low-dose administration versus a short induction course), study design, and sample size. Moreover, CLAD may enter a plateau phase in some patients, with relative stabilization of lung function occurring even in the absence of specific therapeutic interventions. Consequently, the observed attenuation in lung function decline cannot be attributed to MSC therapy and may, at least in part, reflect the natural history of the disease. Furthermore, the absence of a control group and the limited sample size preclude any conclusions regarding therapeutic efficacy. The observed trends therefore provide preliminary observations that warrant further investigation in controlled studies.

Our protocol, based on prolonged low-dose monthly MSC administration, may theoretically allow sustained immunoregulatory activity at the level of the lung allograft, although the biological mechanisms underlying these observations remain to be clarified.

We are aware of the limitations of our study. The small sample size, single-centre compassionate-use design, heterogeneous patient population, and the lack of a control group preclude any conclusions regarding therapeutic efficacy. The inclusion of patients with different CLAD phenotypes, underlying diseases, maintenance immunosuppressive regimens, and disease duration may have influenced treatment response and limits the generalizability of our findings. Furthemore, spontaneous stabilization of lung function has been described in a subset of patients with CLAD and represents a plausible alternative explanation for the observed attenuation in lung function decline. Survivor bias may have influenced the observed longitudinal functional trajectories, as only patients surviving long enough to continue treatment contributed to the longest follow-up observations. Although interrupted time-series analysis strengthens the inference of temporal association, residual confounding cannot be excluded. In addition, the limited sample size also resulted in wide confidence intervals around the infection rate estimates, and the study was therefore underpowered to exclude a clinically meaningful increase in infection risk. Missing pulmonary function data reflected differences in follow-up duration among patients and were handled using mixed-effects models fitted to all available observations without data imputation. Finally, we did not assess biological markers of MSC activity, such as circulating extracellular vesicles, miRNAs or cytokine levels, which could have provided mechanistic insights into the biological effects of prolonged MSC administration. Future research should focus on controlled trials to define optimal dosing, timing, and patient selection, and to evaluate MSC-derived products, such as extracellular vesicles, as potential cell-free therapies.

## 5 Conclusion

Long-term monthly administration of allogeneic, BM-MSCs in advanced CLAD is feasible and appears safe, without increasing the risk of thromboembolism or causing systemic toxicity. Although no statistically significant increase in respiratory infection incidence was detected, the study was underpowered to exclude a clinically meaningful increase in infection risk.

In our cohort, lung function trajectories showed attenuation of functional decline over time. However, given the limited sample size and absence of a control group, these findings should be interpreted cautiously and cannot establish a causal effect of MSC therapy. These findings support the continued exploration of MSCs as a promising therapeutic strategy for CLAD and highlight the need for controlled clinical trials to confirm efficacy.

## Data Availability

The original contributions presented in the study are included in the article/Supplementary Material, further inquiries can be directed to the corresponding author.
