# Supplementary material for: Long-term monthly mesenchymal stromal cell therapy for advanced chronic lung allograft dysfunction: a single-centre safety and feasibility cohort study
Source: Front Med (Lausanne). 2026 Sep 14;13:1942517. doi: 10.3389/fmed.2026.1942517 (PMC13617032; doi:10.3389/fmed.2026.1942517)
Supplement: Supplementary file 1 [file Table1.docx]

| **FEV1** | **Patient 1** | **Patient 2** | **Patient 3** | **Patient 4** | **Patient 5** | **Patient 6** |
| --- | --- | --- | --- | --- | --- | --- |
| - **36 months** | 1,76 | 1,6 | 0,83 | 2,35 | 2,35 | 1,35 |
| - **24 months** | 1,52 | 1,59 | 0,67 | 2,27 | 3,1 | 1,33 |
| - **12 months** | 1,09 | 1,41 | 0,63 | 1,67 | 2,56 | 1,28 |
| **T0** | 0,56 | 1,35 | 0,62 | 1,78 | 0,86 | 1,24 |
| **+ 12 months** | 0,64 | 1,3 | 0,67 | 1,63 | 1,06 | 1,15 |
| **+ 24 months** | 0,65 | 1,23 | 0,49 | 1,58 | 0,99 | 1,23 |
| **+ 36 months** | 0,63 | 1,16 | 0,55 | 1,18 |  |  |
| **+ 48 months** | 0,63 | 1,11 |  |  |  |  |

| **FVC** | **Patient 1** | **Patient 2** | **Patient 3** | **Patient 4** | **Patient 5** | **Patient 6** |
| --- | --- | --- | --- | --- | --- | --- |
| - **36 months** | 2,65 | 3,28 | 1,86 | 2,96 | 2,49 | 1,74 |
| - **24 months** | 2,48 | 3,62 | 1,65 | 2,98 | 3,35 | 1,78 |
| - **12 months** | 2,26 | 3,65 | 1,67 | 2,56 | 2,79 | 1,58 |
| **T0** | 1,09 | 3,1 | 1,64 | 2,78 | 1,74 | 1,71 |
| **+ 12 months** | 1,33 | 3,05 | 1,62 | 2,63 | 2,01 | 1,54 |
| **+ 24 months** | 1,27 | 3,06 | 1,18 | 2,52 | 1,73 | 1,66 |
| **+ 36 months** | 1,24 | 2,94 | 1,29 | 2,43 |  |  |
| **+ 48 months** | 1,14 | 2,55 |  |  |  |  |
